# Increased Expression in Dorsolateral Prefrontal Cortex of CAPON in Schizophrenia and Bipolar Disorder

**DOI:** 10.1371/journal.pmed.0020263

**Published:** 2005-09-13

**Authors:** Bin Xu, Naomi Wratten, Erik I Charych, Steven Buyske, Bonnie L Firestein, Linda M Brzustowicz

**Affiliations:** 1Department of Genetics, Rutgers University, Piscataway, New Jersey, United States of America,; **2**Department of Cell Biology and Neuroscience, Rutgers University, Piscataway, New Jersey, United States of America,; **3**Department of Statistics, Rutgers University, Piscataway, New Jersey, United States of America,; **4**Department of Psychiatry, University of Medicine and Dentistry of New Jersey, New Jersey Medical School, Newark, New Jersey, United States of America; Kings College LondonUnited Kingdom

## Abstract

**Background:**

We have previously reported linkage of markers on chromosome 1q22 to schizophrenia, a finding supported by several independent studies. Within this linkage region, we have identified significant linkage disequilibrium between schizophrenia and markers within the gene for carboxyl-terminal PDZ ligand of neuronal nitric oxide synthase (CAPON). Prior sequencing of the ten exons of *CAPON* failed to reveal a coding mutation associated with illness.

**Methods and Findings:**

We screened a human fetal brain cDNA library and identified a new isoform of *CAPON* that consists of the terminal two exons of the gene, and verified the expression of the predicted corresponding protein in human dorsolateral prefrontal cortex (DLPFC). We examined the expression levels of both the ten-exon *CAPON* transcript and this new isoform in postmortem brain samples from the Stanley Array Collection. Quantitative real-time PCR analysis of RNA from the DLPFC in 105 individuals (35 with schizophrenia, 35 with bipolar disorder, and 35 psychiatrically normal controls) revealed significantly (*p* < 0.005) increased expression of the new isoform in both schizophrenia and bipolar disorder. Furthermore, this increased expression was significantly associated (*p* < 0.05) with genotype at three single-nucleotide polymorphisms previously identified as being in linkage disequilibrium with schizophrenia.

**Conclusion:**

Based on the known interactions between CAPON, neuronal nitric oxide synthase (nNOS), and proteins associated with the N-methyl-D-aspartate receptor (NMDAR) complex, overexpression of either CAPON isoform would be expected to disrupt the association between nNOS and the NMDAR, leading to changes consistent with the NMDAR hypofunctioning hypothesis of schizophrenia. This study adds support to a role of CAPON in schizophrenia, produces new evidence implicating this gene in the etiology of bipolar disorder, and suggests a possible mechanism of action of CAPON in psychiatric illness.

## Abstract 2

Comparative analysis of CAPON expression in postmortem brain samples from patients with schizophrenia, bipolar disorder, and from healthy controls.

## Introduction

Schizophrenia (SCZD) is a serious neuropsychiatric illness estimated to affect approximately 1% of the general population. Family, twin, and adoption studies have demonstrated that schizophrenia is predominantly a genetic disorder, with a high heritability (reviewed in [1]). Multiple genetic and nongenetic factors are likely to be involved [2]. As part of a genome-wide search for loci contributing to risk for schizophrenia, we previously reported linkage, with a maximum heterogeneity lod score of 6.5, to chromosome 1q21-1q22 (SCZD9) in a group of 22 medium-sized Canadian families that were selected for study because multiple relatives were clinically diagnosed with schizophrenia or schizoaffective disorder [3]. We have also reported the results of fine linkage mapping of this 1q21-1q22 region in the same sample of individuals, narrowing the region most likely to harbor this susceptibility locus to approximately 1 Mb between APOA2 and D1S2675, again with a maximum multipoint heterogeneity lod score of 6.5 [4]. Other studies have also reported linkage [5–8] and linkage disequilibrium (LD) [9] of schizophrenia to this region. More recently, we have tested markers from this region for evidence of LD to schizophrenia, identifying significant LD with several markers within the gene for carboxyl-terminal PDZ ligand of neuronal nitric oxide synthase (CAPON; also termed nitric oxide synthase 1 [neuronal] adaptor protein [NOS1AP]) [10]. Association of single-nucleotide polymorphisms (SNPs) within *CAPON* to schizophrenia has recently been replicated in the Chinese Han population [11], although with association detected in the Chinese sample to SNPs located more distal in the gene than the SNPs associated in the Canadian sample.

CAPON is an attractive candidate for schizophrenia susceptibility. CAPON was first identified in the rat as a neuronal nitric oxide synthase (nNOS) binding protein, capable of disrupting the association of nNOS with the postsynaptic density scaffolding proteins PSD93 and PSD95 through the binding of the carboxyl terminus of CAPON to nNOS [12]. The interaction of nNOS with PSD93 and PSD95 is important in targeting nNOS to the postsynaptic N-methyl-D-aspartate receptor (NMDAR) complex and facilitates the tight coupling between activation of the NMDAR and nNOS, allowing nNOS activation by Ca^2+^ influx through the NMDAR and producing NMDAR-mediated NO release into the synaptic structures [13,14]. This places CAPON at the scene of NMDAR glutamate neurotransmission, long proposed to be involved in schizophrenia (reviewed in [15]). CAPON can also serve as an nNOS adaptor protein, with the amino terminus binding either to a direct target of NO-mediated activation by S-nitrosylation [16] or to Synapsin [17], resulting in the localization of nNOS to the presynaptic terminals.

Sequencing of the coding region of *CAPON* in individuals from the Canadian linkage sample failed to identify any coding mutations associated with illness [10], consistent with current results for other candidate genes for schizophrenia [18]. *CAPON* has a large, approximately 300-kb genomic extent, only 1.5 kb of which represents coding sequence. Therefore, there are many potential sites for regulatory sequences that could be disrupted and lead to altered gene expression. In this study, we screened a human cDNA library to identify possible alternative splice forms of *CAPON,* documented *CAPON* mRNA and protein expression in postmortem tissue from the dorsolateral prefrontal cortex (DLPFC) of human brains, and investigated the expression of *CAPON* by quantitative real-time PCR in the Stanley Array Collection, derived from DLPFCs of individuals with schizophrenia and with bipolar disorder, and a set who were psychiatrically normal controls.

## Methods

### Identification and Characterization of CAPON Transcripts

A human fetal brain arrayed cDNA library (Origene Technologies, Rockville, Maryland, United States; #LLFB-1001) was screened using the supplier's protocol. PCR amplification was performed with primers from *CAPON* exon 10 (Ex10-F, 5′- AAATCAACAACCTTGCCTAACG-3′; Ex10-R, 5′- GAAAGCACTCCAGCTTCACC-3′). Exon 10 sequence was chosen for screening purposes to identify alternative transcripts that also contained the nNOS-binding PDZ domain. Individual positive clones were sequenced on a CEQ 8000 (Beckman Coulter, Fullerton, California, United States). The transcript for the previously undescribed short isoform was further characterized by 3′ and 5′ RACE, performed with RACE-ready cDNA from human brain (Ambion, Austin, Texas, United States). For each reaction, a pair of nested PCR primers were designed from short-form 5′ UTR sequences (5RACES, 5′- TTAGAGGTTCCTGGAGGGTGGTGC-3′; 5RACESn, 5′- TTGAGTCCAAGGAGAGGGTAGTGG-3′) and 3′ UTR sequence (3RACE1, 5′- AATGAATGCAAGCTGATAGCTGAGACTG-3′; 3RACE1n, 5′- TGAATCACTGCCACTTGGGTCAGG-3′; 3RACE2, 5′- AGAAGGAAGAACCAGGAAAGTGAGATCC-3′; 3RACE2n, 5′- ATCCAGTGTGGCTGAGCCTACCTAGC-3′; and 3RACE3, 5′- ATGTGGATGGAGAGGGCTTGT-3′; 3RACE3n, 5′- GTGAGGAAGGCCGCTTCTAAAT-3′) and were used in conjunction with a set of universal nested primers (supplied). Products were cloned into TOPO TA cloning vector (Invitrogen, Carlsbad, California, United States) and sequenced on a CEQ 8000.

The sequence for the novel *CAPON* transcript has been deposited in GenBank (http://www.ncbi.nlm.nih.gov/entrez/) under accession number AY841899.

### Human Postmortem Samples

RNA and DNA samples from the Stanley Array Collection of the Stanley Brain Collection (http://www.stanleyresearch.org/programs/brain_collection.asp) were analyzed. This is a collection of biomaterials derived from postmortem brain specimens from 35 individuals with schizophrenia, 35 individuals with bipolar disorder, and 35 psychiatrically normal controls. Diagnoses were made by two senior psychiatrists, applying DSM-IV criteria to medical records, and, when necessary, telephone interviews with family members. Diagnoses of unaffected controls were based on structured interviews by a senior psychiatrist with family member(s) to rule out DSM-IV Axis I diagnoses of psychiatric clinical syndromes.

Specimens were collected, with informed consent from next of kin, by participating medical examiners between January 1995 and June 2002. The specimens were collected, processed, and stored in a standardized way. Exclusion criteria for all specimens included: (1) significant structural brain pathology on postmortem examination by a qualified neuropathologist, or by premortem imaging; (2) history of significant focal neurological signs premortem; (3) history of central nervous system disease that could be expected to alter gene expression in a persistent way; (4) documented IQ under 70; or (5) poor RNA quality. RNA integrity and purity were determined with an Agilent 2100 Bioanalyzer (Agilent, Palo Alto, California, United States). Degradation was defined as a shift in the RNA size distribution toward smaller fragments and a decrease in fluorescence signal of ribosomal peaks. Additional exclusion criteria for unaffected controls included age less than 30 (thus, still in the period of maximum risk) and substance abuse within 1 y of death or evidence of significant alcohol-related changes in the liver.

DNA and RNA from Brodmann's area 46 (in the DLPFC) was available for all individuals in the collection. Genotyping and expression analyses were conducted with the samples coded to keep investigators blind to diagnostic status. After the blind was broken, diagnostic status and a range of clinical variables were provided for analysis. These included gender, race, age at time of death, age of onset, postmortem interval (PMI), brain pH, total brain weight, hemisphere used for RNA extraction, smoking status at time of death (coded as nonsmoking for individuals who smoked previously but had quit), antipsychotic status at time of death, and lifetime antipsychotic exposure in fluphenazine milligram equivalents. In addition, lifetime alcohol and substance use were each rated on a scale of 0 to 5 using the categories “little or none,” “social,” “moderate past,” “moderate present,” “heavy past,” and “heavy present.” Total sample storage time was calculated from date of death to date of experiment, so it represents the sum of storage time of the brain tissue prior to RNA extraction and storage time of the extracted RNA. Overall, the sample was 66% male, with a mean age at death of 44 y (standard deviation [SD] 8.9 y, range 19–64 y), and was predominantly Caucasian (97%). The mean PMI was 33 h (SD 16 h, range 9–84 h), and the mean storage time was 5.0 y (SD 1.9 y, range 2.1–9.7 y). Smoking status at time of death was available for 67 individuals, with 72% of the sample smokers. Lifetime alcohol use estimates were available on all but one patient, with 57% of the sample reporting no, little, or social use, 17% reporting past or present moderate use, and 26% reporting past or present heavy use. Lifetime substance use estimates were available on all but two individuals, with 65% of the sample reporting no, little, or social use, 16% reporting past or present moderate use, and 19% reporting past or present heavy use. The mean age of onset for the schizophrenia group was 21.3 y (SD 6.1 y, range 9–34 y) and for the bipolar group was 25.1 y (SD 9.1 y, range 14–48 y). Further information about the Stanley Array Collection is available from the Stanley Medical Research Institute (http://www.stanleyresearch.org/programs/brain_collection.asp).

Studies of CAPON protein were conducted using brain specimens from five normal control individuals (three male and two female) obtained from the Human Brain and Spinal Fluid Resource Center (Los Angeles, California, United States). Individuals ranged in age from 53 to 90 years at time of death (mean 67.4 y, SD 16.4 y). Samples were recovered and frozen after a mean PMI of 21.5 h (SD 3.0 h, range 17.8–26.0 h). Specimens were shipped frozen for protein and RNA extraction in our laboratory. Mean storage time prior to extraction for these specimens was 3.0 y (SD 1.4 y, range 1.9–5.3 y); protein and RNA was quantified within 1 wk of extraction. To assure a similar composition of each sample for protein and RNA extractions, samples were first ground while still frozen into a fine powder using liquid nitrogen and a mortar and pestle. Aliquots of this homogeneous powder were then used for protein and RNA extraction as below.

### Preparation and Western Blotting of Human Brain Homogenates

Tissue from Brodmann's area 46 (DLPFC) from the Human Brain and Spinal Fluid Resource Center samples was homogenized with 10 times the equivalent volume per weight of TEE (25 mM Tris-HCl [pH 7.4], 1 mM EDTA, 1 mM EGTA, and 1 mM PMSF) using a serrated Teflon pestle. Ten micrograms of protein were loaded and resolved on a 12% SDS polyacrylamide gel and transferred to polyvinylidene difluoride membrane (Immobilon P; Millipore, Billerica, Massachusetts, United States) in transfer buffer lacking SDS, which was determined to interfere with detection of the short isoform of CAPON (CAPON-S). Blots were probed with a rabbit antibody to CAPON (Santa Cruz Biotechnology, Santa Cruz, California, United States; #R-300) and visualized using ECL plus (Amersham Biosciences, Piscataway, New Jersey, United States) with a secondary antibody coupled to horseradish peroxidase. For actin normalization, blots were incubated in strip buffer (62.5 mM Tris-HCl [pH 7.4], 2% SDS, and 90 μM β-mercaptoethanol) at 65 °C for 20 min with agitation, followed by probing with a rabbit antibody to actin (the antigen corresponds to amino acids 20–33, conserved in all actin isoforms; Sigma, Saint Louis, Missouri, United States) using the procedure described above. To ensure that quantification used an exposure that was within the linear range of the film used to image the blot, a series of images with increasing exposure time to the blot were developed. The intensity of the bands on each image was quantified using Image Pro image analysis software (Media Cybernetics, Silver Spring, Maryland, United States) and plotted to determined at what exposure time the darkest band intensities were no longer in the linear range. Nonsaturated exposures were then used to determine the ratio of CAPON to actin. Using ImagePro image analysis software, the relative immunoreactivities of anti-CAPON bands were calculated as *(a* − *b)*/*(c* − *d),* where *a* = sum of pixels in each CAPON immunoreactive band; *b* = sum of pixels in an area equivalent to that occupied by *a* within a nonreactive portion of the lane; *c* = sum of pixels in each actin immunoreactive band; and *d* = sum of pixels in an area equivalent to that occupied by *c* within a nonreactive portion of the lane*.*

### COS-7 Cell Culture and Transfection

COS-7 cells were plated at 70%–80% confluence and maintained in Dulbecco's Modified Eagle Medium (Invitrogen), supplemented with 7.5% fetal bovine serum and 7.5% horse serum in a 7% CO_2_ atmosphere. Cells were transfected with 0.5 μg of plasmid from the Origene library containing the *CAPON* short-form RNA, cloned behind the CMV promoter in pCMV6-XL4, using the LipofectAMINE 2000 method (Invitrogen) following the manufacturer's instructions. After allowing 48 h for expression, cells were lysed in TEE, and lysates were analyzed by Western blotting as described above.

### RNA Extraction

Total RNA was extracted from the Human Brain and Spinal Fluid Resource Center samples using RNeasy Lipid Tissue Mini Kits (Qiagen, Valencia, California, United States), following the manufacturer's instructions. RNA was quantified with a Pharmgena UV spectrometer. Quality of the samples was verified by fractionation on a 2% agarose gel at 100 V for 40 min, staining with ethidium bromide, and visualization under UV light.

### RNA Quantification

Total RNA (5 μg) was treated with the DNA-free Kit (Ambion) in accordance with the manufacturer's protocol to eliminate DNA contamination. The resulting DNase-treated RNA was used in a 40-μl reverse transcriptase reaction to synthesize cDNA following the SuperScript II First-Strand cDNA Synthesis protocol (Invitrogen), including optional RNaseOUT treatment. Samples were then quantified using real-time PCR. The previously described primer pair NN05224/NN05225 (Human Unidentified Gene-Encoded database [http://www.kazusa.or.jp/huge/index.html] clone KIAA0464) was used for quantification of full-length *CAPON* [19,20]. This primer pair produces a 338-bp product that spans the boundary of exons 7 and 8. The primers specific for the short form were designed using Primer Express Software Version 2.0 (Applied Biosystems, Foster City, California, United States). The forward primer (Short-F: 5′- CATTCATGTCCCTCTCTTCTCTC-3′) is located in the 5′ coding sequence that is unique to the short form transcript, the reverse primer (Short-R, 5′- AATGCAGGTCCTCTGGCTTAG-3′) is located within exon 10, and the pair produces a 321-bp product that spans the boundary of exons 9 and 10. The housekeeping gene *ACTB,* the gene encoding beta-actin, was used as reference gene to normalize the total RNA input. The forward (b-actin-F, 5′- CATCCTCACCCTGAAGTACCC-3′) and reverse (b-actin-R, 5′- GAGAAGATGACCCAGATCATGTTT-3′) primers produce a 184-bp product that spans the boundary of exons 3 and 4. Real-time PCR analysis was conducted using 1 μl of a 1:5 dilution of cDNA, 0.1 μM of each primer, and 5 μl of SYBR Green Master Mix (Applied Biosystems) in a total reaction volume of 10 μl in a 384-well plate on an ABI Prism 7900HT sequence detector system (Applied Biosystems). Samples were initially warmed to 50 °C for 2 min, then the AmpliTaq Gold DNA polymerase was activated by heating to 95 °C for 10 min. PCR amplification was performed with 40–50 cycles of 95 °C for 30 s, 58 °C (full-length experiments) or 61 °C (short-form experiments) for 40 s, and 72 °C for 1 min. Each real-time PCR assay was repeated three times. The standard curve used for determining the relative quantity of each isoform in each sample was constructed by the amplification of serial dilutions of pooled brain cDNA. In each experiment, the *R* ^2^ value of the standard curve was greater than 0.98, and the no-template control produced no detectable signal. Dissociation curve analysis was conducted on all PCR products to ensure that only a single product was present in the reaction. Real-time PCR data acquisition and analysis were performed using SDS v2.0 software (Applied Biosystems).

### SNP Genotyping

DNA samples from the Stanley Array Collection were genotyped for three SNPs from dbSNP, rs1415263, rs4145621, and rs2661818, that were previously identified as being in linkage disequilibrium with schizophrenia by a primer extension strategy (Pyrosequencing, Uppsala, Sweden) using the automated PSQ HS96A platform as previously described [10].

### Statistical Analyses

RNA amounts were quantified by the ABI Relative Quantitation of Gene Expression protocol (Applied Biosystems; http://docs.appliedbiosystems.com/pebiodocs/04303859.pdf). The results from three repeat assays were averaged to produce a single mean quantity value for each mRNA for each individual. The quantity values of the target gene were then normalized over the quantity values of the reference gene *ACTB* (encoding beta-actin) to produce normalized expression quantities. These are unitless measures of the relative amount of transcript that is present in each individual.

Normalized expression quantities and patient variables were analyzed with SAS v8.2 for UNIX software (SAS Institute, Cary, North Carolina, United States) and with the R statistical environment [21]. Correlation between protein and mRNA levels in control brain samples from the Human Brain and Spinal Fluid Resource Center was tested with Pearson's product moment correlation. For the samples from the Stanley Array Collection, potentially confounding variables (Table 1) were tested for association with *CAPON* expression in all individuals. As *CAPON* expression levels correlated with time of storage, subsequent analyses included storage time as a covariate. Effect of exposure to antipsychotics (either as a dichotomous trait or a quantitative estimate of total lifetime exposure) was examined only in patients with bipolar disorder or schizophrenia. Continuous variables (age at death, postmortem interval, brain pH, and brain weight) were tested by regression; dichotomous variables (gender, brain hemisphere analyzed, smoking status at time of death, and history of exposure to antipsychotic medication) and ordinal variables (lifetime alcohol use and lifetime substance abuse) were tested by ANOVA. The strength of the association with *CAPON* expression was measured with Pearson's product moment correlation for continuous variables (except for quantitative lifetime antipsychotic exposure, for which Spearman's correlation was used) and Kendall's rank correlation for binary and ordinal variables. The relationship between gene expression levels and diagnostic group was summarized with Kendall's rank correlation and tested with ANOVA, and the relationship between gene expression levels and age of onset within the bipolar and schizophrenia groups was summarized with Pearson's product moment correlation and tested with regression. The relationship between genotype and *CAPON* expression was analyzed using ANOVA with a term for years of storage (as in all of the ANOVAs), a term coded for a dominance effect of the SNP, and a term for a recessive effect of the SNP.

**Table 1:**
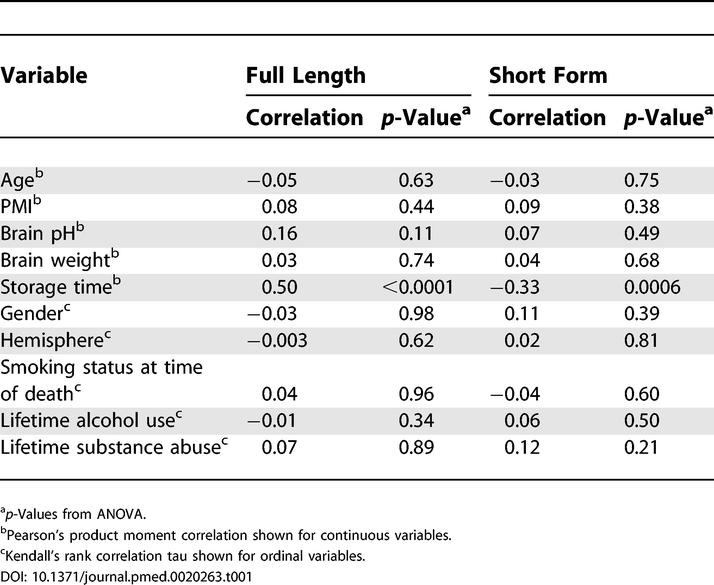
Correlations between *CAPON* Isoform Expression and Possible Confounding Variables

## Results

### Identification and Characterization of Two Human CAPON Isoforms

Screening of a human fetal brain cDNA library resulted in the isolation of four distinct clones. Three clones (“*CAPON* full-length”) contained inserts of approximately 2.5 kb, corresponding to exons 1–10, as previously described in the NCBI reference sequence for *CAPON* [12,22], except that exon 4 from these clones was missing the first 15 bp listed in the reference sequence. The fourth clone (“*CAPON* short-form”) contained an insert of 4 kb with a unique 5′ untranslated region (UTR) corresponding to genomic sequence from intron 8, followed by exons 9 and 10, and a 3′ UTR longer than that contained by the other clones or in the reference sequence. This transcript is predicted to produce a 211 amino acid protein, including 18 novel amino acids at the amino terminus, and will contain the CAPON PDZ-binding domain.

The previously undescribed short-form transcript of *CAPON* was further characterized by 5′ and 3′ RACE. The 5′ UTR was 2,367 bp, while the longest 3′ RACE product ended 2,556 bp downstream of the stop codon. From these results, the total length of the short-form mRNA was calculated to be 5,559 bp. Due to the much longer 5′ and 3′ UTRs, the *CAPON* transcript encoding the short-form of the protein is actually significantly longer than the transcript that encodes the full-length protein. The gene, transcripts, and predicted protein structures of the two forms are shown in Figure 1.

**Figure 1:**
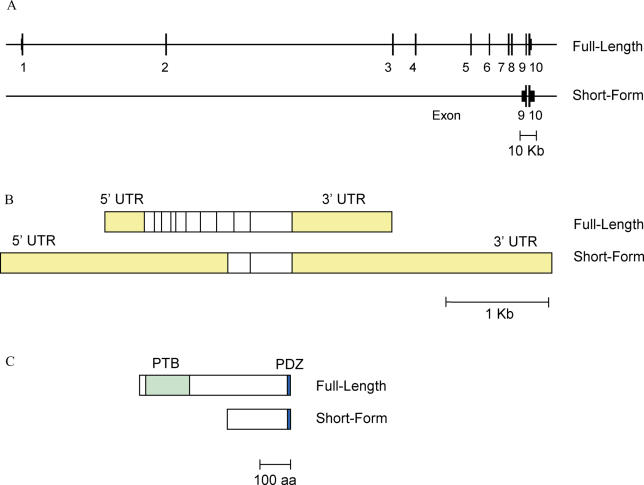
CAPON Gene Structure, Isoforms, and Protein Functional Domains CAPON full-length transcript and protein illustrations are based on NCBI reference sequences. (A) Genomic organization of *CAPON*. Exons are represented by numbered boxes; 5′ and 3′ UTR are represented by half-height boxes. (B) *CAPON* transcripts. 5′ and 3′ UTR are represented by yellow shaded boxes, exons by white boxes. (C) CAPON proteins. The phosphotyrosine-binding domain (PTB) is shaded green, and the PDZ-binding domain is shaded blue.

Protein and total RNA were extracted from Brodmann's area 46 (DLPFC) of postmortem samples from five normal controls, COS-7 cell that had been transfected with cDNA encoding the CAPON short-form, and untransfected COS-7 cells that normally express the CAPON long form. Western blots of these samples were probed with a rabbit polyclonal antibody raised against the carboxyl terminus of CAPON. This antibody is predicted to interact with both the full-length and short-form of CAPON, since the antigen used to generate the antibody is in the carboxyl termini of both forms. Bands were observed at the expected sizes, near the 75-kDa marker for the full-length protein and near the 30-kDa marker for the short-form (Figure 2). There appears to be two smaller forms of the full-length CAPON (CAPON-L bracket, Figure 2), which could be due to posttranslational modification (i.e., phosphorylation) of CAPON. For example, analysis by software designed to identify potential sites for phosphorylation by protein kinase C (http://mpr.nci.nih.gov/mpr/ScanProteinForPKCSitesPage.aspx) identified five such sites in the full-length CAPON sequence. The short form of CAPON appears as a doublet, although the presence of the lower band (CAPON-S′) in untransfected COS-7 cells may indicate that this band is caused by recognition by the CAPON antibody of a cross-reacting protein. The appearance of the higher band (CAPON-S) in the transfected cells, which clearly comigrates with a band in human brain tissue, indicates that the short-form transcript is translated into a protein of the expected size in DLPFC.

**Figure 2:**
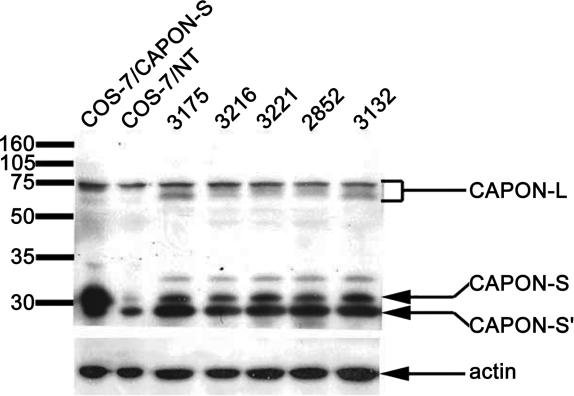
Western Blot of CAPON Protein Isoforms in DLPFC from Normal Control Individuals Tissue from Brodmann's area 46 (DLPFC) from five individuals was homogenized in TEE. Proteins were resolved by SDS-PAGE and transferred to PVDF membrane. Blots were probed with rabbit polyclonal antibodies to CAPON and actin, and proteins were detected using chemiluminescence. Band intensities for CAPON-L, CAPON-S, and CAPON-S + CAPON-S′ were calculated and normalized to the intensities of the corresponding actin bands. Untransfected COS-7 cells expressing CAPON-L and CAPON-S′ (COS-7/NT) and COS-7 cells expressing recombinant CAPON-S (COS-7/CAPON-S) and were used as controls for these proteins. CAPON-L appears to include multiple bands, possibly due to phosphorylation.

As protein samples were not available for the Stanley Array Collection samples, we tested the correlation between protein and RNA expression using the normal control brain samples to determine if RNA levels could be used as a reasonable indicator of protein expression for CAPON. CAPON protein levels were quantified from Western blot image analysis and were normalized to levels of actin protein, while RNA levels were quantified by reverse-transcription real-time PCR normalized to levels of *ACTB* (beta-actin). For full-length CAPON, the correlation between protein and RNA levels was significant (*p* = 0.019) with *r* = 0.94. For the short form, the correlation was also significant (*p* = 0.0049) with *r* = 0.97 between the RNA and protein levels of the S band, which corresponds to the size of the cloned CAPON product. While it seems likely that the S′ band represents a cross-reacting protein, given its presence in untransfected COS-7 cells, it is possible that it could represent a modified form of the short-form protein. Considering the CAPON short-form product as the sum of the S and S′ bands, the correlation with RNA levels remained significant (*p* = 0.030), with *r* = 0.91.

### Analysis of CAPON Isoform Expression by Diagnosis

Expression levels of both *CAPON* isoforms were determined by reverse-transcription real-time PCR for all 105 samples from the Stanley Array Collection. Expression levels were normalized to *ACTB* (beta-actin), and these normalized relative expression levels were used for all subsequent analyses. No significant correlations were detected between mRNA levels of either *CAPON* isoform and the potentially confounding variables of age at death, PMI, brain pH, brain weight, gender, hemisphere, smoking status at time of death, lifetime alcohol use, or lifetime substance abuse (Table 1). *CAPON* expression levels were found to be significantly (*p* < 0.001) correlated with length of sample storage for both isoforms (Table 1). Therefore, for all subsequent analyses storage time was used as covariate.

The potential confounding effects of antipsychotic medication treatment on *CAPON* expression levels were also very important to examine, but since all of the patients with schizophrenia and none of the controls had been treated with such medications, the effects of treatment and diagnosis could not be separated by analyses that included these groups. Within the 35 individuals in the bipolar group, however, 18 individuals were on antipsychotic medication at the time of death, 11 individuals had never received antipsychotic medication, and six individuals were not on antipsychotic medication at time of death, but had been treated with these medications at some point in the past. *CAPON* levels were compared between antipsychotic-treated and untreated individuals with bipolar disorder. Neither a positive history of lifetime antipsychotic use nor antipsychotic use at time of death was significantly correlated with *CAPON* short-form expression within the bipolar group (Table 2). In contrast, expression of full-length *CAPON* was significantly correlated with treatment (Table 2), with a 40% decrease in mean expression in the patients (*n* = 24) with bipolar disorder and a history of treatment with antipsychotics in the past or at time of death (*p* = 0.003), and a 45% decrease in patients (*n* = 18) receiving antipsychotics at time of death (*p* = 0.0007), when compared to antipsychotic-untreated individuals (*n* = 11) with bipolar disorder. An estimate of total lifetime antipsychotic medication was available for all but one individual in the bipolar and schizophrenia groups with a positive history of antipsychotic treatment (*n* = 58). No significant correlations were found between levels of lifetime antipsychotic exposure and expression of either *CAPON* isoform (Table 2).

**Table 2:**
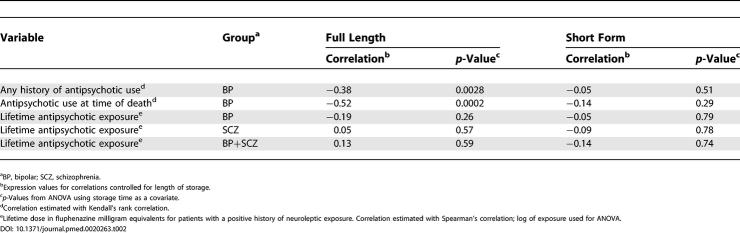
Effect of Antipsychotic Treatment on *CAPON* Isoform Expression

Overall, there was no significant difference in *CAPON* full-length mRNA expression across diagnostic categories (Figure 3). Since treatment with antipsychotics may influence expression of the full-length isoform, we examined expression of this transcript in antipsychotic-naïve patients with bipolar disorder. While mean *CAPON* full-length mRNA levels were increased by 24% in patients with bipolar disorder but no history of exposure to antipsychotic medication (*n* = 11) as compared to normal controls, this increase did not reach statistical significance (*p* = 0.11). Results were similar when comparing bipolar patients not receiving antipsychotic medication at time of death, regardless of past treatment history, (*n* = 17) to normal controls, with a 18% increase in full-length *CAPON* levels (*p* = 0.14).

**Figure 3:**
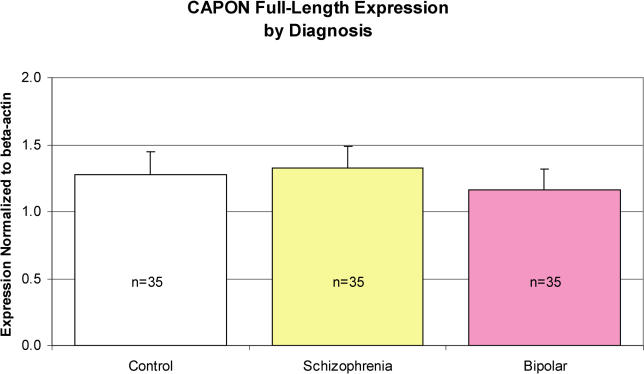
ACTB (Beta-Actin)–-Normalized *CAPON* mRNA Full-Length Expression by Diagnosis Expression levels are least squares means. Mean values per category are plotted with 95% confidence intervals. The number of individuals per sample is indicated within each bar. Level of expression does not differ significantly by diagnostic group. The mean (95% confidence interval lower bound, upper bound) for the control, schizophrenia, and bipolar groups are 1.28 (1.12, 1.45), 1.33 (1.17, 1.49), and 1.16 (0.99, 1.32), respectively.

Mean *CAPON* short-form mRNA levels were significantly increased by 48% in the schizophrenia group (*p* = 0.0035) and 50% in the bipolar group (*p* = 0.0002) as compared to the control group (Figure 4). The schizophrenia and bipolar groups did not differ significantly from each other in *CAPON* short-form expression (*p* = 0.94). *CAPON* short-form expression was significantly correlated with the age of onset in the schizophrenia group (Pearson's *r* = 0.53, *p* = 0.0008), but not in the bipolar group (*r* = −0.02, *p* = 0.92). This significance (or lack thereof) is unchanged when age of death is included as a covariate. The majority of samples were from individuals of European decent (97%), with one African American individual with bipolar disorder, one Native American individual with bipolar disorder, and one Hispanic individual with schizophrenia. None of these individuals exhibited extreme values for expression of either *CAPON* isoform, and re-analysis with these patients excluded did not change which comparisons reached statistical significance (unpublished data).

**Figure 4:**
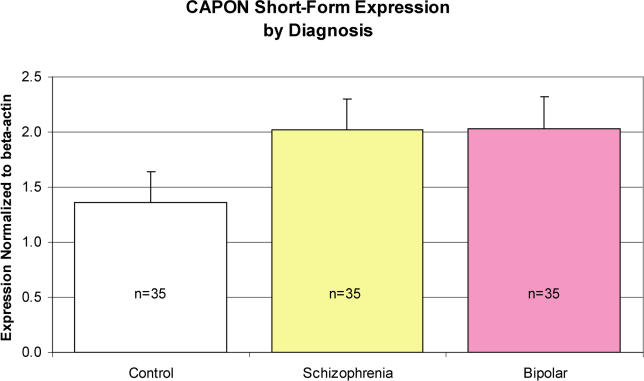
ACTB (Beta-Actin)–Normalized *CAPON* mRNA Short-Form Expression by Diagnosis Expression levels are least squares means. Mean values per category are plotted with 95% confidence intervals. The number of individuals per sample is indicated within each bar. Expression is significantly higher in patients with schizophrenia (*p* = 0.0013) and bipolar (*p* = 0.0009) as compared to controls. The mean (95% confidence interval lower bound, upper bound) for the control, schizophrenia, and bipolar groups are 1.34 (1.05, 1.62), 2.02 (1.73, 2.30), and 2.05 (1.77, 2.34), respectively.

### Analysis of CAPON Isoform Expression by Genotype

All individuals were genotyped at rs1415263, rs4145621, and rs2661818, three SNPs within *CAPON* that were previously identified as being in significant linkage disequilibrium with schizophrenia [10]. For each SNP, individuals with one or two copies of the previously identified associated allele were observed to have higher group mean *CAPON* short-form expression than the group of individuals homozygous for the unassociated allele (Figure 5). All three SNPs individually showed significant differences among means for the short-form expression (rs1415263, *p* = 0.019; rs4145621, *p* = 0.022; rs2661818, *p* = 0.019), while none showed significantly different means for the full-length expression (rs1415263, *p* = 0.67; rs4145621, *p* = 0.52; rs2661818, *p* = 0.50). Genotypes with one or two copies of the associated alleles had higher mean short-form expression (rs1415263, 30%; rs4145621, 32%; rs2661818, 34%). None of the SNPs showed significant expression differences between individuals heterozygous or homozygous for the associated allele. Given that the prior demonstrated correlation between *CAPON* full-length expression and antipsychotic treatment could represent a medication treatment effect, the correlation analysis between *CAPON* full-length expression and genotype was rerun using only individuals not receiving antipsychotic medications at time of death (35 controls and 17 individuals with bipolar disorder). Again, there were no significant differences in mean *CAPON* full-length expression among genotypes for any of these SNPs (rs1415263, *p* = 0.41; rs4145621, *p* = 0.82; rs2661818, *p* = 0.58).

**Figure 5:**
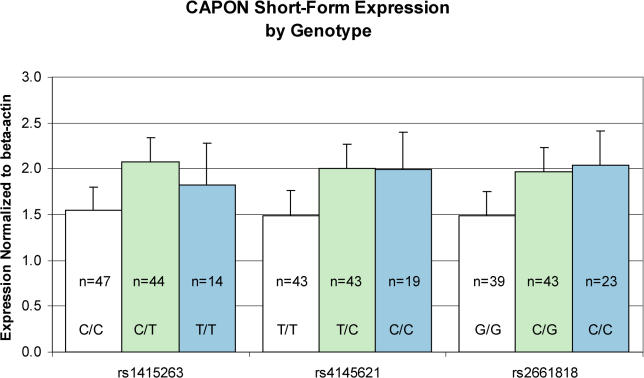
ACTB (Beta-Actin)–Normalized *CAPON* mRNA Short-Form Expression by Genotype Expression levels are least squares means. Individuals from all three diagnostic classifications are included, grouped only by genotype. Mean values per genotype for each SNP are plotted with 95% confidence intervals. The number of individuals per genotype is indicated within each bar. SNP alleles are given for forward strand sequence. All three SNPs exhibit significantly (*p* < 0.05) different levels of *CAPON* expression by genotype, with a dominant effect. Higher levels of *CAPON* are seen in individuals with one or two copies of alleles previously identified as associated with schizophrenia (T, rs1415263; C, rs4145621; and C, rs2661818). The mean (95% confidence interval lower bound, upper bound) for the three genotypes for each SNP are as follows. For rs1415263: 1.54 (1.29, 1.80) for CC; 2.07 (1.81, 2.34) for CT; and 1.83 (1.36, 2.29) for TT. For rs4145621: 1.51 (1.25, 1.78) for TT; 2.01 (1.74, 2.27) for TC; and 2.01 (1.61, 2.41) for CC. For rs2661818: 1.49 (1.21, 1.76) for GG; 1.96 (1.70, 2.23) for CG; and 2.04 (1.68, 2.41) for CC.

## Discussion

Our screening of a human fetal total brain cDNA library resulted in the identification of two isoforms of *CAPON* mRNA corresponding to two forms of CAPON protein. Our screen used only primers from exon 10, so we would not have detected isoforms lacking this portion of the gene. One of the two identified transcripts encompasses ten exons and encodes a 501 amino acid protein containing two known functional domains, an amino-terminal phosphotyrosine-binding domain and a carboxyl-terminal PDZ-binding domain. This full-length form corresponds to transcripts previously identified in the rat and human [12,22]. The second transcript contains the last two exons of *CAPON* and is predicted to produce a short form of the protein, 211 amino acids long and containing the PDZ-binding domain. Prior work has demonstrated that the terminal 125 amino acids of the full-length protein are sufficient to bind the PDZ-domain of nNOS and interfere with the binding between nNOS and PSD93 or PSD95 [12]. In addition to the ability of CAPON to bind to nNOS, the terminal 125 amino acids also appear to be able to directly bind to the second PDZ domain of PSD95 [23], the normal site of nNOS binding to PSD95 [13]. As the first 180 amino acids of CAPON have been previously demonstrated to contain the domain needed to bind to the amino-terminal targets Dexras1 and Synapsin [16,17], it would seem that only the full-length form of CAPON would be able to serve as an adaptor protein between nNOS and these targets. A physiological role of the short form would likely be limited to the competitive inhibition of binding of other ligands to the PDZ domains of nNOS and PSD93 or PSD95.

There are significant obstacles to the study of gene expression in the human brain. Obtaining high-quality postmortem samples suitable for RNA extraction is difficult and labor-intensive. Obtaining appropriate matched control groups is also a challenge. While it may be possible to collect samples with relative consistency across some variables, such as PMI or brain pH, many factors that may potentially affect gene expression, such as treatment history and substance use, are beyond the control of investigators. The rate of collection of individuals with too many clinical restrictions (e.g., treatment-naïve individuals with schizophrenia and no history of substance abuse, alcohol use, or smoking) would be too slow to produce a useful number of samples. Added to these clinical variables is likely etiological heterogeneity, with only a subset of affected individuals expected to harbor a primary causative mutation in any given gene. All of these factors may lessen the chance that significant differences in gene expression can be demonstrated using a particular sample.

We chose to conduct our expression studies using the Stanley Array Collection, as this collection contains samples from more individuals than other postmortem collections, and the samples were collected in a standardized fashion with an emphasis on obtaining high-quality RNA for expression studies. Limitations of this collection include the facts that protein samples were not available for parallel analysis, and that only one brain region, the DLPFC, was available for study. However, this brain region has long been hypothesized to be involved in schizophrenia, implicated by evidence from neuropsychological, neuroimaging, histopathological, and neurochemical studies (reviewed in [24]).

Our results suggest that mRNA expression of the short-form of *CAPON* is significantly (*p* < 0.005) increased in patients with either schizophrenia or bipolar disorder. If the short-form protein behaves as predicted, it would disrupt the binding of nNOS to PSD95 through competitive inhibition and remove nNOS from the NMDAR complex, thereby decoupling NO generation from NMDAR activation. This could produce a picture consistent with the NMDAR hypofunction hypothesis of schizophrenia. Based on our data, expression of short-form mRNA does not appear sensitive to treatment with antipsychotic medication. Full-length *CAPON* mRNA expression, in contrast, appears to be highly influenced by treatment with antipsychotic medication, at least in bipolar disorder. It will be of interest to further investigate and confirm this effect in subsequent studies. Pre- and postexposure expression studies in animals may be helpful in determining if the relationship between antipsychotic treatment and decreased *CAPON* mRNA expression is causal. While we found no significant group differences in expression levels between patients with schizophrenia or bipolar disorder and normal controls, it is possible that this is due to the normalization of full-length *CAPON* mRNA expression by antipsychotic treatment. Additional expression studies in individuals with schizophrenia not receiving antipsychotic medication would be of great interest to assess this possibility.

The Stanley Array Collection consists of samples collected from several locations within the United States, and therefore represents a sample that is independent from our Canadian familial schizophrenia collection. Nonetheless, there is significant evidence for association between affection phenotypes and the same alleles at three different SNPs in both samples. Consistent with the hypothesis that *CAPON* short form overexpression is associated with schizophrenia, the alleles observed associated with schizophrenia in our Canadian sample are significantly (*p* < 0.05) associated with higher short form expression in the Stanley Array Collection.

The three SNPs investigated span nearly 98 kb and are located in introns 2 and 3 of *CAPON,* the most proximal being 70 kb upstream from the short-form transcription start site. Although there is evidence for LD spanning large regions within *CAPON,* it is unlikely that these SNPs are in tight disequilibrium with polymorphisms in the short-form basal promoter [10,25]. Our prior work on this gene revealed that the three SNPs used in this study are in significant (*p* < 0.0001) linkage disequilibrium with each other (rs1415263 and rs4145621, D′ = 0.748; rs1415263 and rs2661818, D′ = 0.801; rs4145621 and rs2661818, D′ = 0.491), while being in much weaker LD (D′ values ranging from 0.074 to 0.432) with SNPs located in intron 8 (rs7521206) and exon 9 (rs348624) [10]. More probable is that the SNPs used in the present study are in LD with a mutation in an enhancer region that is located at some distance upstream of the short-form transcript. Enhancers can regulate gene expression from distances of up to 1 Mb [26], and mutations in enhancer sequences have been shown to be responsible for a number of human diseases [27].

Additional studies are needed to further examine the level of CAPON protein among individuals with different psychiatric diagnoses, in both the DLPFC and other regions of the brain. The implication that CAPON may influence schizophrenia susceptibility through disruption of NMDAR functioning adds to the list of candidate genes that may act at this receptor system, including Neuregulin 1 [28], D-amino acid oxidase and G72 [29], Dysbindin [30], and PPP3CC [31]. Additional work on the interaction of these different candidates may also further our understanding of the genetic component of schizophrenia susceptibility.

## Accession Numbers

The Online Mendelian Inheritance in Man (http://www.ncbi.nlm.nih.gov/entrez/query.fcgi?db=OMIM) accession numbers for some loci discussed in this paper are *SCZD* (181500) and *SCZD9* (604906). The GenBank (http://www.ncbi.nlm.nih.gov/Genbank/index.html) accession numbers for other loci and proteins discussed in this paper are *ACTB* (NM_001101), *APOA2* (X02619), *CAPON/NOS1AP* (RNA, NM_014697; protein, NP_055512), and *D1S2675* (Z52679).

**Patient Summary**

**Background**

Although the exact cause of schizophrenia remains unknown, people who inherit certain variations in genes are thought to be at higher risk. Scientists have been trying to get a handle on which genes are involved. They hope that this will help them understand the disease better and eventually lead to treatments and maybe even prevention. So far, a number of genes have been implicated, but it is important to confirm such findings, and to find out what the genes actually do that could lead to abnormalities in the brain.

**Why Was This Study Done?**

The scientists who did this study had previously discovered that a gene called *CAPON* might be involved in schizophrenia. The scientists had found that certain variations in the region of the gene were more common in patients with schizophrenia. In this study, the researchers examined *CAPON* in more detail and asked two main questions: (1) Are there differences in the activity of the *CAPON* gene in brains from patients with schizophrenia and bipolar disorder compared with those from healthy individuals? (2) Could they confirm the link between variations in the *CAPON* gene and the disease in a second study of different patients with schizophrenia?

**What Did the Researchers Do and Find?**

They looked for variations in the *CAPON* gene and measured the level of *CAPON* gene expression in samples from a collection of postmortem brain specimens. The collection contained brain tissue samples from 35 patients with schizophrenia, 35 patients with bipolar disorder, and 35 patients without psychiatric illness (known as “controls”). Genes are templates for proteins (which make up the majority of active components in cells and body), and the researchers found that the *CAPON* gene is a template for two different proteins, a short form and a long form. Brain samples from patients with schizophrenia and bipolar disorder had higher levels of the short form than brain samples from patients without psychiatric illness. Moreover, these higher levels of the short version were predominantly seen in people with versions of the *CAPON* gene that had been previously linked to schizophrenia.

**What Does This Mean?**

These results lend more support to the idea that *CAPON* is somehow involved in schizophrenia. Given that studies by other groups also point to a link between CAPON and schizophrenia, it seems clear that further study of *CAPON* is justified. The findings here suggest that we need to learn more about the short version of *CAPON,* and specifically what exactly the CAPON protein does in the brain. Because of the limitations of working with human postmortem samples, it is likely that most of the next round of experiments will be done in cell culture and in animal models.

**Where Can I Find More Information Online?**

The following Web sites provide information on schizophrenia.

US National Institutes of Mental Health (search for “schizophrenia”): http://www.nimh.nih.gov/

UK National Institute for Health and Clinical Excellence pages on schizophrenia: http://www.nice.org.uk/page.aspx?o=42770

The National Alliance for Research on Schizophrenia and Depression (NARSAD): http://www.narsad.org/

The National Alliance for the Mentally Ill (NAMI):

http://www.nami.org/

The Schizophrenia Society of Canada:

http://www.schizophrenia.ca/

## Abbreviations

CAPON: carboxyl-terminal PDZ ligand of neuronal nitric oxide synthase
DLPFC: dorsolateral prefrontal cortex
LD: linkage disequilibrium
NMDAR: N-methyl-D-aspartate receptor
nNOS: neuronal nitric oxide synthase
PMI: postmortem interval
SD: standard deviation
SNP: single-nucleotide polymorphism
UTR: untranslated region
